# Supplementary material for: Raloxifene Protects Oxygen-Glucose-Deprived Astrocyte Cells Used to Mimic Hypoxic-Ischemic Brain Injury
Source: Int J Mol Sci. 2024 Nov 12;25(22):12121. doi: 10.3390/ijms252212121 (PMC11594051; doi:10.3390/ijms252212121)
Supplement: Supplementary file 1 [file ijms-25-12121-s001.zip › ijms-3043953-supplementary.pdf]

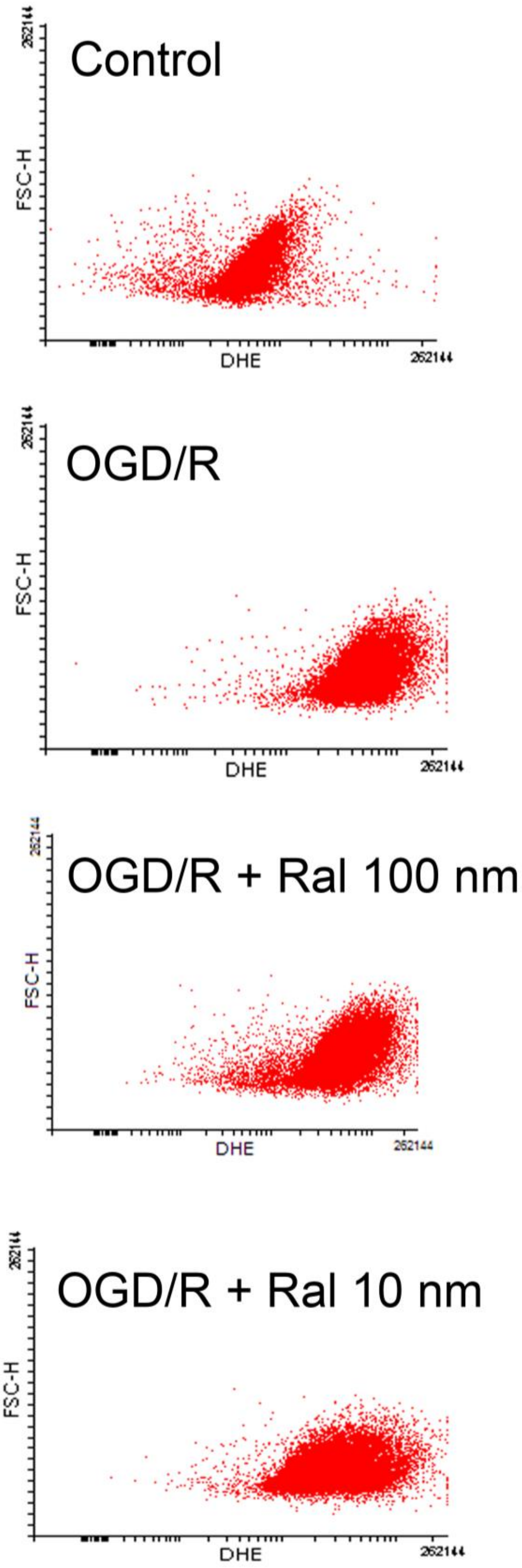

Supplementary Figure S1. Representative flow cytometry plots are shown. Data are represented as the mean ± SEM of 4 independent experiments. Control (5028.58 ± 424.80); OGD/R (39,510.50 ± 1718.64); OGD/R + 100 nM raloxifene (28,734.70 ± 1856.91); OGD/R + 10 nM raloxifene (28,538.67 ± 1017.65). Data underwent analysis of variance, the post hoc Dunnet's test for between-group comparisons, and Tukey's test for multiple comparisons.

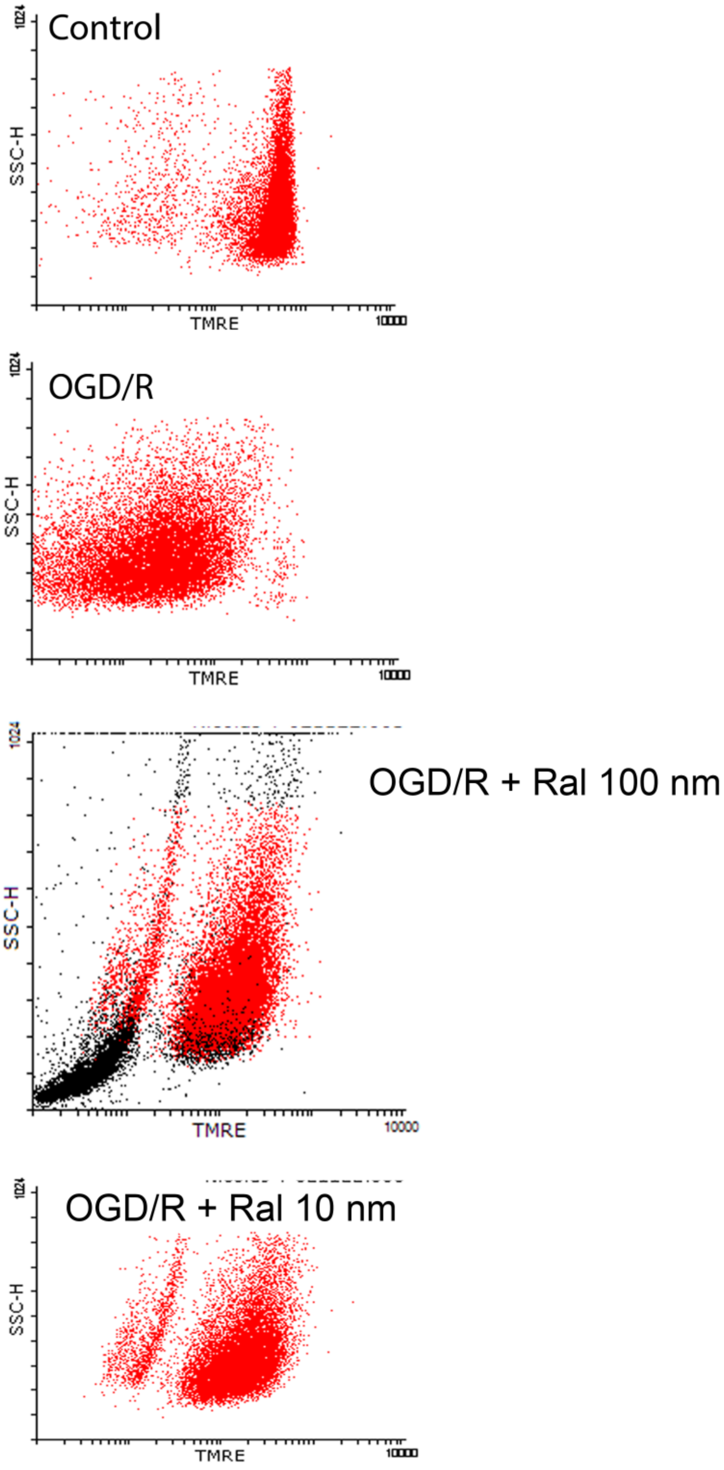

Supplementary Figure S2. Representative flow cytometry plots of tetramethylrhodamine methyl ester (TMRM) intensity. Data are represented as the mean ± SEM of four independent experiments. Control (102.60 ± 7.43); OGD/R (9.86 ± 2.88); OGD/R + 100 nM raloxifene (60.44 ± 14.92); OGD/R + 10 nM raloxifene (83.70 ± 16.84); Carbonyl cyanide 3-chlorophenylhydrazone (CCCP) (24.58 ± 13.58). Data were analysed by variance analysis, followed by the post hoc Dunnet's test for between-group comparisons and Tukey's test for multiple comparisons.
